# Genetic predisposition of suicidal behavior: variants in *GRIN2B*, *GABRG2*, and *ODC1* genes in attempted and completed suicide in two Balkan populations

**DOI:** 10.1007/s00406-024-01895-9

**Published:** 2024-09-19

**Authors:** Jelena Karanović, Doroteja Beraković, Mojca Katrašnik, Iris Šalamon Arčan, Maja Pantović-Stefanović, Lana Radenković, Nemanja Garai, Maja Ivković, Dušanka Savić-Pavićević, Tomaž Zupanc, Alja Videtič Paska

**Affiliations:** 1 Center for Human Molecular Genetics, Faculty of Biology, University of Belgrade, Studentski trg 16, PO box 43, Belgrade, 11000 Serbia; 2 Laboratory for Molecular Biology, Institute of Molecular Genetics and Genetic Engineering, University of Belgrade, Vojvode Stepe 444A, Belgrade, 11042 Serbia; 3 Department of Biotechnology, University of Rijeka, Radmile Matejčić 2, Rijeka, 51000 Croatia; 4 Institute of Biochemistry and Molecular Genetics, Faculty of Medicine, University of Ljubljana, Vrazov trg 2, Ljubljana, 1000 Slovenia; 5 Clinic for Psychiatry, University Clinical Centre of Serbia, Pasterova 2, Belgrade, 11000 Serbia; 6 University of Belgrade-Medical School, Doktora Subotića 8, Belgrade, 11000 Serbia; 7 Institute of Forensic Medicine, Faculty of Medicine, University of Ljubljana, Korytkova ulica 2, Ljubljana, 1000 Slovenia

**Keywords:** Suicidal behavior, Genetic variant, Single nucleotide polymorphism, Association

## Abstract

Completed suicide accounts for over 700,000 deaths worldwide annually, while attempted suicide is 20 times more frequent. Genetic background is an important factor contributing to suicidal behavior, including candidate genes in glutamate, γ-aminobutyric acid (GABA), and polyamine systems. Our aim was to differentiate genetic predispositions underlying different types of suicidal behavior, attempted and completed suicide, in two Balkan populations. Analysis of variants in the genes *GRIN2B* (rs2268115 and rs220557), *GABRG2* (rs424740), and *ODC1* (rs1049500 and rs2302614) was performed on a study sample including 173 suicide attempters with comorbid psychiatric disorders, 216 non-suicidal psychiatric patients and 172 healthy controls from Serbia, and 333 suicide completers and 356 non-suicidal autopsy controls from Slovenia. CA genotype of rs220557 in *GRIN2B* gene increased the risk for completed suicide (*P* = 0.021), and violent suicide (*P* = 0.037), compared to controls. In *ODC1* gene, CA genotype of rs2302614 decreased the risk for completed suicide compared to suicide attempt (*P* = 0.012). Marginally, AC haplotype for variants rs1049500-rs2302614 in *ODC1* gene decreased the risk for completed suicide compared to suicide attempt (*P* = 0.052). Specific genetic variants of glutamate and polyamine systems are differently distributed among diverse suicidal phenotypes, providing further information on the implication of these systems in suicidality.

## Introduction

Suicidal behavior, a continuum ranging from suicidal ideation to completed suicide, is a serious public health problem requiring multilevel diagnostic and therapeutic approaches. Completed suicide accounts for over 700,000 deaths worldwide per year, with a rate of 10.7/100,000 people, while attempted suicide is reported even 20 times more often. A load of suicidal behavior burden extends further to millions of people who suffer from suicidal thoughts, grief, or are in any other way affected by this non-communicable disease [1]. Even though the sex ratio in suicidal patients is different among countries, completed suicide rate is 2.3 times greater in males [2], while attempted suicide is more often in women [3]. Globally, the most common suicidal methods are pesticide self-poisoning, firearms, and hanging [1], while in Europe the most prevalent method is hanging [4].

Suicidal behavior is a complex phenotype, resulting from an interplay of various environmental, sociodemographic, and biological factors [5, 6]. General genetic heritability estimates for suicidal behavior ranges from few percent to up to 55% [7–9]. Heritability of suicide attempt in large cohorts has been calculated to account for ~ 4% [10]. Notably, the strongest predictor for completed suicide is previously attempted suicide, with approximately 40% of completed suicides with a history of attempted suicide [5].

Among the prominent biological findings of suicidal behavior has been the change in the metabolism of neurotransmitter serotonin – the decrease of 5-hydroxyindolacetic acid in the cerebrospinal fluid of depressed suicides [11]. Since then, many candidate genes from various neurotransmitter systems, besides mostly investigated serotonin system, have been implicated in the etiology of suicidal behavior including the glutamate, γ-aminobutyric acid (GABA) and polyamine candidate genes [12].

Glutamate and GABA play a considerable role in central integration of stress responses through hypothalamo-pituitary-adrenocortical (HPA) axis [13]. Glutamate neurons and synapses clearly exceed all other neurotransmitter systems in the brain, and are regulated by GABA neurotransmitter system [14]. The alternations in primary excitatory and inhibitory neurotransmitter systems, glutamate and GABA, respectively, have been demonstrated in etiologies like depression [15, 16] and schizophrenia [17], which are often comorbid with suicidal behavior. Furthermore, chronic stress conditions have been associated with decrease in the structure and function of glutamate neurons and in GABA markers in the brain [18]. Neurobiological alternations of the two main neurotransmitter systems can be supported also with genetic studies.

Glutamate receptors can be divided to metabotrophic and fast acting ionotropic, with the latter being further divided to N-methyl-D-aspartate receptors (NMDAR) and non-NMDAR [19]. Changes in global gene expression in brain tissue samples of (depressed) suicides where shown for glutamine synthetase (involved in glutamate recycling), and glutamate ionotropic and metabotropic receptors, with the latter being typically upregulated [20]. Comparison of suicides with major depressive disorder to non-suicide patients again showed that the expression of glutamate receptors (*GRIN2B*,* GRIK3* and *GRM2*) was higher in suicides [21]. It is of further interest that NMDAR antagonist, ketamine, has been implicated in rapid decrease of suicide ideation sensation [22, 23]. Furthermore, studies on DNA variations reported association of ionotropic receptor GRIN2B with suicide attempt [24].

Genes for receptors, transporter and glutamate-ammonia ligase of the major inhibitory neurotransmitter GABA have been also implicated in suicidality. GABAergic genes, particularly GABAA receptor subunits have been found upregulated in depressed suicides compared to non-suicidal patients [20, 25]. On the other hand, GABAA receptor’s subunit gamma 2 (GABRG2) had lower brain expression in suicide, with further association of the variant rs424740 with suicide death [26]. Other variants of the same gene (rs183294 and rs209356; C-A haplotype) have been associated with a history of suicide attempt in schizophrenia patients with a history of alcohol dependence or abuse [27].

In research of suicidal behavior the polyamine system is of particular interest due to its direct effect on NMDA and non-NMDA receptors, with glutamate receptors containing GRIN2B subunit being the most sensitive to changes in levels of polyamine concentration. The polyamine stress response system seems to be regulated differently when considering suicidal behavior, as well. Polyamines interact with DNA, RNA and lipids, giving them visible roles in regulation of gene transcription and posttranscriptional modifications. Moreover, they are essential for cell proliferation and modulate ion channels and synaptic activity. The levels of the polyamines are regulated through two key enzymes for synthesis and degradation, ornithine decarboxylase 1 (ODC1) and spermidine/spermine N1-acetyltransferase 1 (SAT1), respectively. Imbalance of polyamine metabolism (e.g. the ODC1 overactivity or deficits in the SAT1) might result in hyperexcitation, and in long-term result in changes of neuronal properties [28]. Rather comprehensive amount of studies on suicide have investigated and determined altered levels of the polyamines [29] and gene expression [30]. Genetic variants of both ODC1 [24] and SAT1 [31, 32] have been investigated in suicidal behavior, and provided mixed results.

Studies of genetic variants are still one of the important approaches in identification of biomarkers of many complex, multifactorial disorders. To the best of our knowledge, we performed the first study to differentiate genetic predispositions underlying different types of suicidal behavior, attempted and completed suicide, in two Balkan populations, both with high suicide rates. In 2019 Slovenia ranked on the 7th place, while Serbia on the 25th place in suicidal statistics [33]. We analyzed variants in the candidate genes for NMDA receptor’s subunit (*GRIN2B* rs2268115 and rs220557), for GABAA receptor’s subunit (*GABRG2* rs424740), and for ornithine decarboxylase 1 (*ODC1* rs1049500 and rs2302614).

## Materials & methods

### Subjects

The study sample from Serbian population included 561 unrelated subjects: 173 suicide attempters (SA), 216 suicide non-attempters (non-SA), and 172 healthy controls (HC). All subjects were recruited in the period 2006–2017 from the Department of Psychiatry at the Clinical Centre of Serbia in Belgrade. SA and non-SA groups included psychiatric patients diagnosed with major depressive disorder, bipolar disorder, or schizophrenia according to the Structured Clinical Interview for DSM-IV Axis I Disorders [34] by two trained psychiatrists. Patients from SA group were hospitalized for five weeks immediately after suicide attempt, whereas those from the non-SA group were hospitalized due to recurrence of the primary psychiatric disorder and without history of suicide attempt. Subjects from HC group voluntarily underwent the same procedure as the psychiatric patients. After a detailed discussion of the aims and requirements of the study, written informed consent was obtained from all subjects from Serbian population to participate in the study. All subjects were able to give informed consent. The personal rights of the subjects were respected. The study was approved by the Ethics Committee of the Clinical Centre of Serbia (No. 3672/10) in accordance with the Declaration of Helsinki.

From Slovenian population 689 subjects were included in this research: 333 suicide completers (SC) and 356 non-suicidal autopsy controls (AC). Among SC subjects, 284 used a violent method (such as hanging, colliding with vehicle, use of shotguns, jumping form heights, self-immolation) (group VSC), while 49 people applied non-violent method for suicide competition (such as drowning, substance poisoning and gas poisoning) (group non-VSC). AC group was composed of people that died from sudden cardiac death or by accident, such as in traffic or at work. Samples were collected during autopsy at the Institute of Forensic Medicine, Faculty of Medicine, University of Ljubljana in the period 2014–2016. They were archive samples fixed by formalin and embedded in paraffin. The Commission of the Republic of Slovenia for Medical Ethics assessed that this research is ethically acceptable (No. 0120–436/2021/4).

The demographic characteristics, including age and sex, and data regarding psychiatric diagnoses of the studied subjects from Serbian and Slovenian populations are presented in Tables 1 and 2, respectively.

**Table 1:**
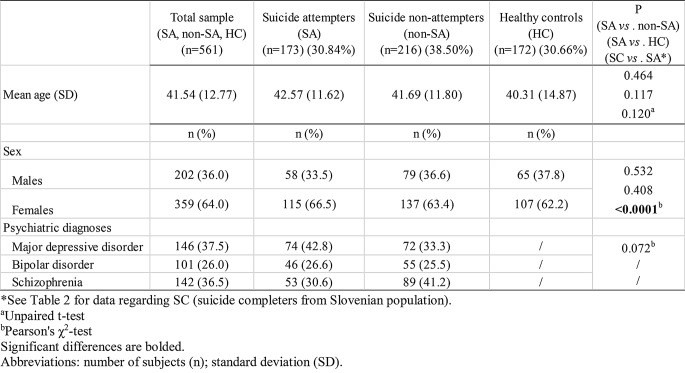
Demographic characteristics and psychiatric diagnoses in the examined sample of Serbian population

**Table 2:**
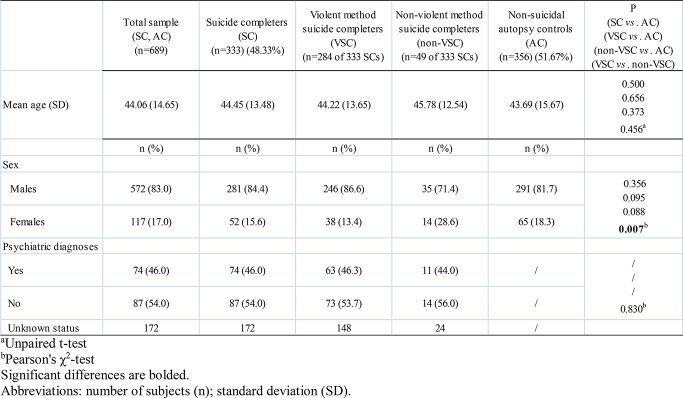
Demographic characteristics and psychiatric diagnoses in the examined sample of Slovenian population

### Genotyping

Blood samples were taken from Serbian population, while archive formalin-fixed paraffin-embedded (FFPE) tissues of liver, kidney, pancreas or heart from Slovenian population. Genomic DNA was isolated from blood samples using the QIAamp DNA Blood Mini Kit (Qiagen, Hilden, Germany), while from FFPE tissues using the Chemagic FFPE DNA Kit H96 (PerkinElmer, Baesweiler, The Netherlands) and Chemagic 360 Instrument (PerkinElmer, Baesweiler, The Netherlands), according to the manufacturers’ protocols. Concentration and purity of isolated DNA from FFPE tissues were analyzed with a Synergy H4 Hybrid Reader spectrophotometer (BioTek, Winooski, Vermont, USA) using a Take3 Plate. Genotyping of rs2268115, rs220557, rs424740, rs1049500, and rs2302614 was performed by allelic discrimination assay using TaqMan^®^ Pre-Designed SNP Genotyping Assays (Applied Biosystems, Warrington, UK), C___2682138_1_, C___2680546_1_, C___3169566_10, C___3265467_10 and C__25597411_30, respectively, following manufacturer’s instructions. Quantitative real-time polymerase chain reactions (RT-qPCR) were performed using StepOnePlus™ Real-Time PCR System (Applied Biosystems, Warrington, UK) in Serbia, and ViiA™ 7 system (Applied Biosystems, Warrington, UK) in Slovenia. 10% of randomly selected samples were genotyped in duplicate with a 100% concordance.

### Statistical analyses

The difference in the subjects’ mean age was tested using unpaired t-test, while the differences in sex ratio and psychiatric diagnoses between studied groups using Pearson’s χ^2^-test, on the GraphPad QuickCalcs Web sites https://www.graphpad.com/quickcalcs/ttest1.cfm and https://www.graphpad.com/quickcalcs/contingency1/ (accessed April 2023), respectively.

All subsequent applied tests were implemented in PLINK version 1.9 [35, 36] (www.cog-genomics.org/plink/1.9/). Genetic variants were tested for Hardy-Weinberg equilibrium only in HC, while other groups included patients and therefore were excluded from this analysis. Pearson’s χ^2^-test or Fisher’s exact test (in case of sample size lower than 5) were applied to test allelic associations, while logistic regression was used to test genotype and haplotype associations with attempted and completed suicide. For two comparisons, VSC vs. non-VSC and SC vs. SA, logistic regression was adjusted by sex due to observed statistically significant differences in sex ratio between these groups (Tables 1 and 2). The significance level was set to *P* < 0.05 in all analyses. The odds ratio (OR) with 95% confidence interval (CI) were used as a measure of the strength of an association. Calculation of the statistical power of the study was performed with G*Power 3.1 Software [37]. For the *P*-value of 0.05, small effect size (0.2), and sample size for Serbian population *n* = 561 and for Slovenian population *n* = 689, the calculated power (1-β) was 0.992 and 0.998, respectively, for χ^2^-test with df = 2. Thus, the study had adequate sample size and statistical power for all statistical analyses.

## Results

Statistically significant differences in sex ratio were observed between SC and SA (χ2 = 133.183, df = 1, *P* < 0.0001, Pearson’s χ^2^-test), as well as between VSC and non-VSC groups (χ2 = 7.319, df = 1, *P* = 0.007, Pearson’s χ^2^-test) (Tables 1 and 2). More females attempted suicide than males in Serbian population (66.5% vs. 33.5%). On the other hand, more males completed suicide than females in Slovenian population (84.4% vs. 15.6%). Among completed suicide in Slovenian population, more males selected violent method than females (86.6% vs. 13.4%), as well as the non-violent method (71.4% vs. 28.6%). However, differences in sex ratio among VSC and non-VSC groups were statistically significant, and thus adjusted by in the following statistical analyses. Altogether, the violent method was more prevalent for both sexes since 87.5% of SC males used violent method, as well as 73.1% of SC females (data not presented in the Table 2). There were no other significant differences in mean age and sex ratio between studied groups (Tables 1 and 2). HC group was demonstrated to be in Hardy-Weinberg equilibrium for all five studied genetic variants (*P* > 0.05, Pearson’s χ^2^-test).

None of five studied genetic variants showed statistical significance in allelic association with attempted or completed suicide (*P* > 0.05, Pearson’s χ^2^-test/Fisher’s exact test) (Table 3). However, three statistically significant genotypic associations with attempted or completed suicide, all involving general (2df) genetic model, were observed for two genetic variants, rs220557 in *GRIN2B* gene, and rs2302614 in *ODC1* gene (*P* < 0.05, logistic regression analyses) (Table 4).

**Table 3:**
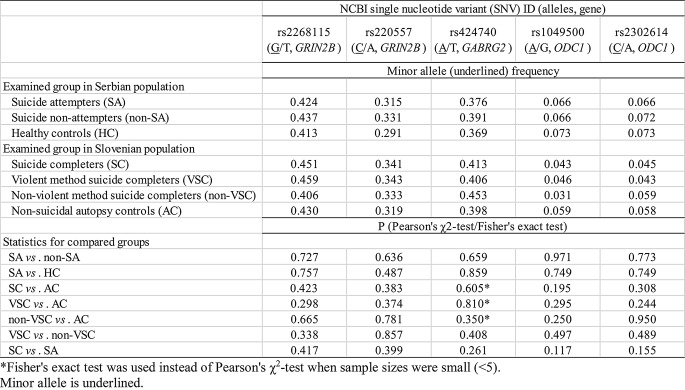
Minor allele frequencies of examined *GRIN2B*, *GABRG2* and *ODC1* genetic variants in suicide patients and controls from Serbian and Slovenian population and their association with attempted and completed suicide

**Table 4:**
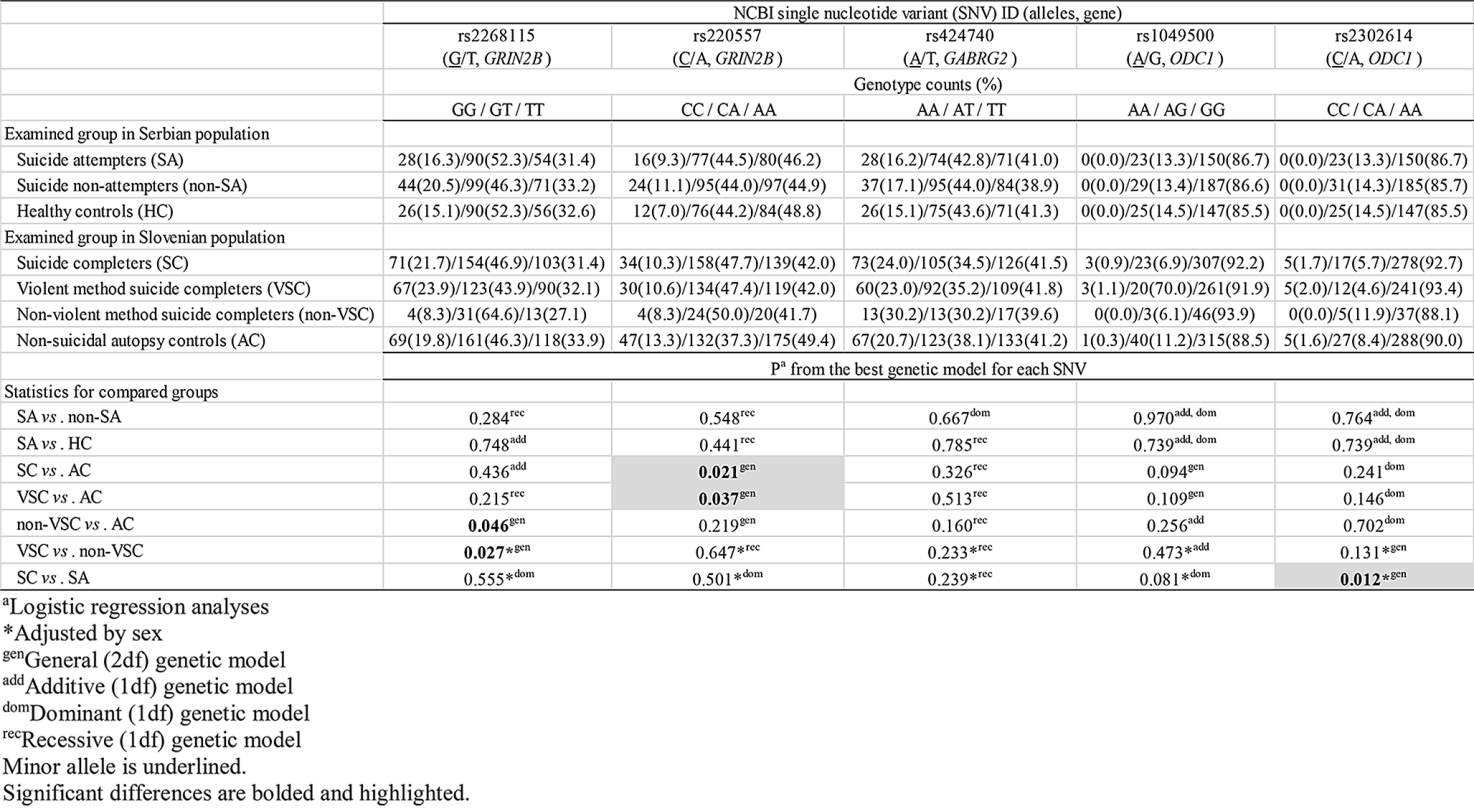
Genotypic counts and percentages of examined *GRIN2B*, *GABRG2* and *ODC1* genetic variants in suicide patients and controls from Serbian and Slovenian population and their association with attempted and completed suicide

For rs2268115 in *GRIN2B* gene, confidence intervals included 1 and therefore these results could not be interpreted as significant (Table 5)

**Table 5:**
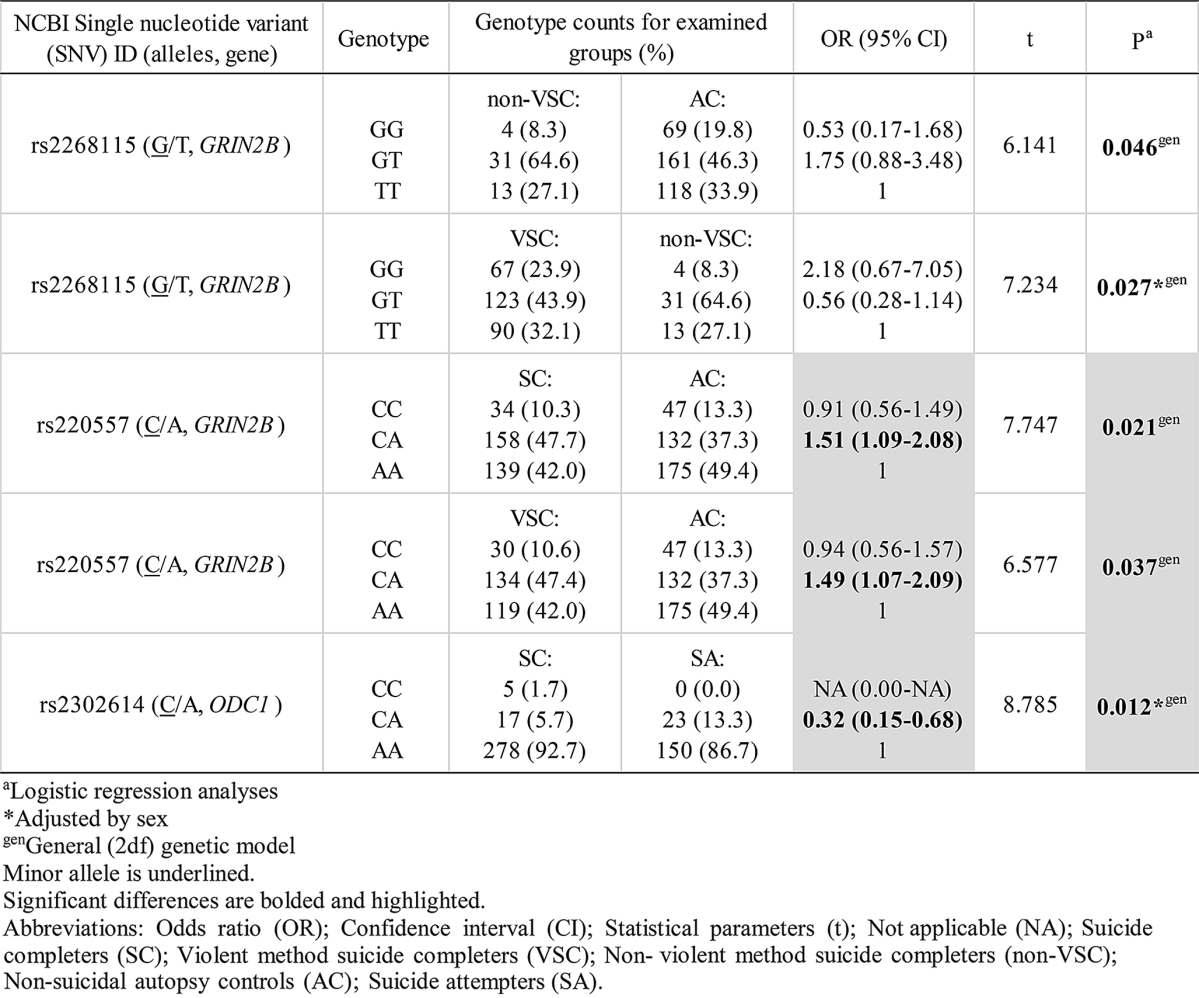
Significant associations of *GRIN2B* and *ODC1* genetic variants with attempted and completed suicide in suicide patients and controls from Serbian and Slovenian population

CA genotype of rs220557 in *GRIN2B* gene increased the risk for completed suicide (OR = 1.51, t = 7.747, *P* = 0.021, logistic regression analysis), and particulary violent suicide (OR = 1.49, t = 6.577, *P* = 0.037, logistic regression analysis), compared to controls (Table 5)

In *ODC1* gene, CA genotype of rs2302614 decreased the risk for completed suicide compared to attempted suicide (OR = 0.32, t = 8.785, *P* = 0.012, logistic regression analysis adjusted by sex) (Table 5). Notably, CC genotype of rs2302614 might, on the contrary, elevated the risk for completed suicide, but odds ratio (OR) could not be calculated since none CC carrier was detected among suicide attempters (Table 5)

Furthermore, in *ODC1* gene, examination of haplotype rs1049500-rs2302614 associations with suicidal behavior revealed marginal significance of AC haplotype in decreasing the risk for completed suicide compared to suicide attempt (OR = 0.50, F = 0.046, t = 3.79, *P* = 0.052, logistic regression analysis adjusted by sex) (Table 6)

**Table 6:**
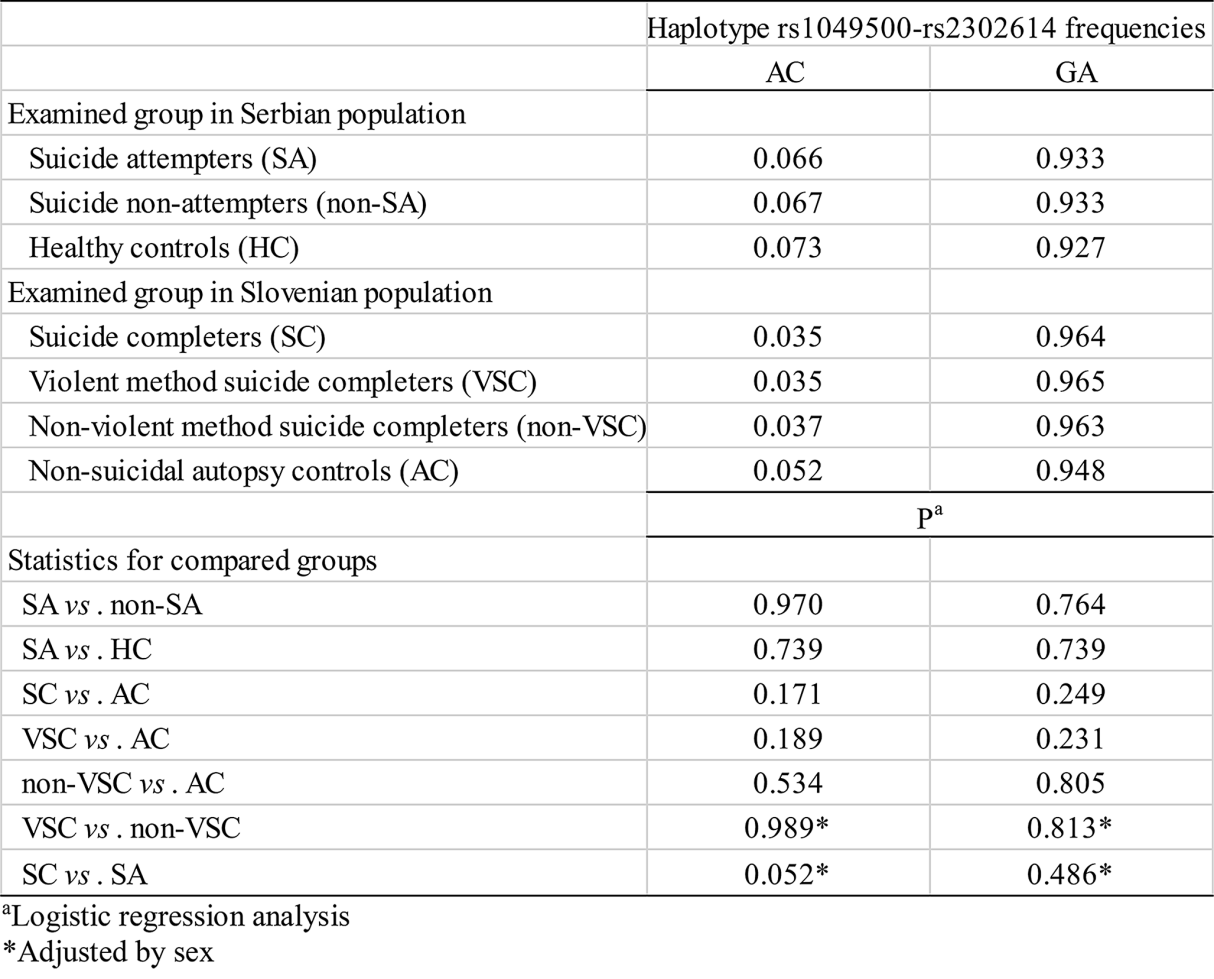
Haplotype frequencies in *ODC1* gene in suicide patients and controls from Serbian and Slovenian population and their association with attempted and completed suicide

## Discussion

In this study, we report the results of the investigation of five variants from three candidate genes, *GRIN2B*, *GABRG2* and *ODC1*, on two types of suicidal behavior, attempted and completed suicide from two Balkan countries, Serbia and Slovenia. For *GRIN2B* gene, rs220557 CA carriers were at risk for completed suicide, and particularly for violent completed suicide, compared to controls. For *ODC1* gene, rs2302614 CA genotype decreased the risk for completed suicide compared to attempted suicide. Finally, AC haplotype for the variants rs1049500-rs2302614 in the *ODC1* gene marginally decreased the risk for completed suicide in comparison to attempted suicide, as well.

Globally, more males complete suicide [2], while more females attempt suicide [3]. Our results support this distinct sex ratio representation for attempted and completed suicide. Among suicide attempters, 66.5% were females, while among suicide completers, 84.4% were males. Regarding suicide methods, violent methods are overrepresented, with hanging being the most prevalent in European populations of both sexes [4]. For Slovenian population we had the data regarding used suicide method. In our study, violent methods were used in 85.3% of total suicidal victims (87.5% males and 73.1% females), and as in other European populations, suicide by hanging was the most frequently used method among both sexes, followed by firearms and drowning, respectively. Similar data was observed in previous studies in Slovenia [38]. For Serbian suicide attempters data on suicide methods were not available, so we could not perform direct comparison. From the literature, hanging is also the most prevalent method in Serbian population [39].

Suicidal behavior, including both, attempted and completed suicide, are associated with extensive neurobiological pathways alternations in the brain. The extent of changes that is associated with one or the other type of suicidality remains elusive, due to the complex nature of these phenotypes [5]. Interrogation of the selected genetic variants has been done before, but never simultaneously on both types of suicide behavior in genetically close populations. In our study, CA carriers of the rs220557 in *GRIN2B* gene were at risk for suicide and violent suicide compared to controls. In a family-based study the variants of *GRIN2B* gene (rs1805247-rs1806201-rs1805482-rs2268115 AGGC haplotype) were observed to be linked with suicide attempt, rs2268115 and rs220557 were associated with alcohol-drug use disorders and the use of violent methods among female suicide attempters, as well as with higher anger among suicide attempters [24]. The rs2268115 allele G was more frequent in alcohol-dependent patients with suicide attempt than in non-attempters with a twofold increase in odds [40]. Other variants of the *GRIN2B* gene have been associated with treatment resistant depression [41].

Polyamines modulate a broad range of neurotransmitter receptors and ion channels, including NMDA. They comprise the polyamine stress response system, which can function even independently of the HPA axis. Studies showed that in acute or chronic stress state the levels of ODC1 elevate [30]. The abovementioned family-based study linked suicide attempt with *ODC1* rs1049500 and rs2302614 variants, as well [24].

The strengths of the study are simultaneous research of attempted and completed suicide, which enables comparison of genetic background of distinct types of suicidal behavior. Inclusion of healthy controls is important for comparison against psychiatric patients with and without suicide attempt, to reveal the association of genetic variants with suicide specifically. Although the study includes two related Balkan populations with high suicide rate, some limitations could be identified. We did not include a permutation test since it was not applicable for a general genetic model in PLINK. For suicide completers the history for psychiatric diagnosis was not available, but we compensated this with revision of medico-legal autopsy documentation regarding existing medical status and toxicological analysis for ethanol and centrally acting drugs. Since the subgroups with identified psychiatric diagnosis and/or positive toxicological analysis were relatively small the effect of genetic variants was not calculated. Therefore, the interaction of psychiatric diagnosis and suicide could not be observed, even though the psychiatric diagnosis is evidenced as strong suicidal risk factor [5].

## Conclusion

Our study showed association of the genetic variants of the brain’s major excitatory pathway and its modulator, the GRIN2B and ODC1, respectively, with attempted and completed suicide, suggesting different genetic backgrounds of two suicidal types. Although analyses of candidate genes seem to be outdated compared to the high throughput genome-wide association studies, interrogating hundreds of thousands of variants throughout the genome simultaneously, the candidate gene approach still contributes to better understating of intricate genetic background of suicidality phenotypes. In the future, more attention would be welcome to improve the study design, inclusion of more detailed phenotypes and sufficient sample sizes to gain the statistical power to detect small effect sizes.
